# The Stevens–Johnson Syndrome/Toxic Epidermal Necrolysis Spectrum: A Report of Two Contrasting Cases Managed in a Burn Unit

**DOI:** 10.3390/diseases14100355

**Published:** 2026-09-25

**Authors:** Mihai-Codrin Constantinescu, Dan-Cristian Moraru, Vladimir Poroch, Stefana Avadanei-Luca, Andrei-Nicolae Gologan, Alexandru-Hristo Amarandei, Awad Dmour, Dragos-Florin Gheuca-Solovastru, Bianca-Ana Dmour, Delia-Gabriela Ciobanu-Apostol, Mihaela Pertea

**Affiliations:** 1 “Grigore T. Popa” University of Medicine and Pharmacy, 700115 Iasi, Romania; mihai-codrin.constantinescu@umfiasi.ro (M.-C.C.); vladimir.poroch@umfiasi.ro (V.P.); stefana_luca@umfiasi.ro (S.A.-L.); andrei-nicolae.gologan@d.umfiasi.ro (A.-N.G.); alexandru-hristo.amarandei@d.umfiasi.ro (A.-H.A.); dmour-awad@umfiasi.ro (A.D.); mg-eng-31249@students.umfiasi.ro (D.-F.G.-S.); bianca-ana.dmour@umfiasi.ro (B.-A.D.); delia.ciobanu@umfiasi.ro (D.-G.C.-A.); mihaela.pertea@umfiasi.ro (M.P.); 2 Department of Plastic Surgery and Reconstructive Microsurgery, Sf. Spiridon Emergency County Hospital, 700111 Iasi, Romania; 3 Cardiology Clinic, Sf Spiridon Emergency County Hospital, 700111 Iasi, Romania

**Keywords:** Stevens-Johnson syndrome, toxic epidermal necrolysis, clinical diagnosis, IVIg, corticosteroids, re-epithelialization

## Abstract

Background: Stevens–Johnson syndrome (SJS) and toxic epidermal necrolysis (TEN) are severe, potentially life-threatening mucocutaneous reactions classified based on body surface area (BSA) involvement: SJS affects less than 10% BSA, SJS/TEN overlap affects 10–30% BSA, and TEN affects more than 30% BSA. The mortality rates range from 4.8–9% for SJS to 14.8–48% for TEN. Treatment remains controversial, with corticosteroids and intravenous immunoglobulin (IVIg) being the most debated therapeutic options. While some studies suggest IVIg may block Fas-mediated keratinocyte apoptosis, meta-analyses have shown conflicting results regarding mortality benefit. Case presentation: We describe two patients from opposite ends of the SJS/TEN spectrum, managed in a burn unit with different therapeutic approaches. The first patient, a 47-year-old man with systemic lupus erythematosus, developed TEN with approximately 35% body surface area involvement, complicated by sepsis and rhabdomyolysis; he was treated with systemic corticosteroids and intravenous immunoglobulin (IVIg, 0.4 g/kg/day for 3 days). The second patient, a 66-year-old woman, developed SJS attributed to amoxicillin–clavulanic acid and was treated with systemic corticosteroids alone. Both patients achieved complete re-epithelialization without major sequelae. Conclusions: These two cases illustrate the clinical heterogeneity of the SJS/TEN spectrum and the central role of early recognition, prompt withdrawal of the culprit drug, transfer to a specialized unit, and multidisciplinary supportive care. Given the substantial differences between the two patients in age, sex, comorbidities, culprit drugs, and disease severity, no comparative or causal inference regarding the relative efficacy of the two treatment strategies can be drawn; no efficacy claim is made for either therapeutic approach; this report is descriptive, and the two cases are presented as contrasting rather than comparable.

## 1. Introduction

Stevens–Johnson syndrome (SJS) was first described in 1922 by American pediatricians Albert Mason Stevens and Frank Chambliss Johnson, who reported two children presenting with “an extraordinary, generalized eruption with continued fever, inflamed buccal mucosa and severe purulent conjunctivitis” [1]. Toxic epidermal necrolysis (TEN), also known as Lyell syndrome, was first described by Alan Lyell in 1956 as an eruption resembling scalding of the skin [1]. Currently, SJS and TEN are considered to represent a spectrum of the same disease process, differing primarily in severity and extent of epidermal detachment [2]. The classification scheme, originally proposed in 1993 by an international panel of experts, defines these conditions based on the percentage of body surface area (BSA) with epidermal detachment [2]:

- Stevens–Johnson syndrome (SJS): epidermal detachment affecting less than 10% of BSA [2,3,4,5].
- SJS/TEN overlap: epidermal detachment affecting 10–30% of BSA [2,3,4].
- Toxic epidermal necrolysis (TEN): epidermal detachment affecting more than 30% of BSA [2,3,4].

SJS and TEN are rare conditions with reported incidence rates varying by geographic location. The incidence of SJS ranges from 1 to 7 cases per million population per year, while TEN occurs at a rate of 0.4 to 1.5 cases per million per year [2,6,7,8]. The estimated pediatric incidence is 5.3 cases per million children per year for SJS and 0.4 cases per million children per year for TEN [3,7]. Regarding gender distribution, females are more commonly affected than males at a ratio of approximately 1.5:1 [3]. The gender distribution for SJS and TEN is almost equal, with slightly more females affected, while a female preponderance of approximately 65% has been observed in SJS/TEN overlap [6].

The mortality rates differ substantially across the disease spectrum: 4.8–9% for SJS, 19.4–29% for SJS/TEN overlap, and 14.8–48% for TEN [3,4]. According to the German population-based registry, mortality is almost 10% for patients with SJS, approximately 30% for patients with SJS/TEN overlap, and almost 50% for patients with TEN, with an overall mortality rate of approximately 25% for all categories combined [6].

Both conditions are characterized by widespread epidermal necrosis, mucocutaneous involvement, and are considered dermatologic emergencies with significant morbidity and mortality [2,3]. The clinical presentation typically begins with a prodromal phase of 1–3 days, characterized by fever, malaise, and upper respiratory symptoms, followed by the acute phase with painful erythematous macules that rapidly progress to flaccid blisters and widespread epidermal detachment [2,3]. A hallmark clinical finding is the positive Nikolsky sign, elicited by applying tangential mechanical pressure to perilesional or apparently uninvolved skin, which causes the epidermis to separate and slide off the underlying dermis [2,3]. This sign reflects the loss of keratinocyte adhesion due to extensive apoptosis. Although characteristic of SJS/TEN, the Nikolsky sign is not specific, as it may also be positive in other blistering disorders such as pemphigus vulgaris and staphylococcal scalded skin syndrome; the diagnosis therefore relies on the overall clinical and histopathological context. Mucosal involvement, affecting the oral, ocular, and genital mucosae, occurs in over 90% of patients and often precedes cutaneous manifestations [2,3].

Several risk factors have been identified for the development of SJS/TEN. The incidence rises with increasing age and is at least 1000-fold higher in HIV/AIDS patients [2]. Patients with systemic lupus erythematosus (SLE) may also be at higher risk, although this may reflect the greater number of medications prescribed to these patients [2]. Immunocompromised patients demonstrate elevated incidence and mortality rates, with risk correlating to worsening immune function [8]. The pathogenesis of SJS/TEN is not yet fully understood, but clinical and histopathological studies indicate that these conditions represent a form of delayed-type hypersensitivity reaction, primarily triggered by drugs [2].

In 2008, the critical cytotoxic role of granulysin in SJS/TEN was demonstrated [3]. Granulysin is a pro-apoptotic protein produced by cytotoxic T cells and NK cells that permits cell-mediated cytotoxicity without direct cell-to-cell contact. In SJS/TEN blisters, granulysin levels are significantly elevated, with concentrations two to four orders of magnitude higher than other cytotoxic proteins such as perforin, granzyme B, or soluble FasL [6]. The severity of cutaneous lesions correlates with serum granulysin levels [4]. Beyond granulysin, drug-specific cytotoxic CD8+ T lymphocytes, restricted by specific HLA class I alleles, are recognized as central to the immunopathogenesis of SJS/TEN [9,10].

The vast majority of SJS/TEN cases are drug-induced, with medications accounting for approximately 75–80% of cases in TEN and 50–80% in SJS [2,3,11,12]. The remaining cases are attributed to infections, vaccinations, or remain idiopathic [3,11]. Medications associated with moderate risk include cephalosporins, macrolide antibiotics, quinolone antibiotics, tetracyclines, and acetic acid-type NSAIDs such as diclofenac [5,11]. The latency period between initiation of high-risk drugs and onset of cutaneous reaction typically ranges between 4 and 28 days, with more than 90% of cases occurring within the first 63 days of drug use [6,11]. For drugs intended for long-term use, the risk is elevated primarily during the initial two months of treatment [2]. Other infectious triggers include herpes simplex virus and, rarely, hepatitis A virus and human herpesvirus 7 [3]. The management of SJS/TEN remains challenging due to the rarity of the condition and the consequent lack of large-scale randomized controlled trials. In fact, randomized controlled trials in SJS/TEN remain very scarce: an early trial of thalidomide versus placebo was halted because of excess mortality in the thalidomide group [2,11], and, more recently, a randomized controlled trial compared the TNF-α antagonist etanercept with corticosteroids, reporting a shorter skin-healing time with etanercept [13]. Consequently, treatment recommendations, including the 2024 German S3 guideline, are largely based on retrospective studies, case series, and expert opinion [14]. Transfer to specialized units, such as burn centers or intensive care units, is recommended for patients with extensive skin involvement, as prompt referral has been shown to reduce infection rates and mortality [2,15].

The role of systemic corticosteroids remains controversial. Earlier studies suggested that corticosteroid treatment increased the risk of infection, delayed re-epithelialization, prolonged hospitalization, and increased mortality [4,16]. However, more recent European multicenter retrospective studies and meta-analyses have suggested beneficial effects, particularly when high-dose corticosteroids are administered early in the disease course [4]. Short-term use of corticosteroids in the early stages may reduce mortality without significantly increasing infection risk, provided adequate wound management and infection control measures are implemented [4]. Intravenous immunoglobulin (IVIg) has been widely used based on the hypothesis that antibodies in pooled human IVIg block Fas receptor-mediated keratinocyte apoptosis [11]. Cyclosporine, a calcineurin inhibitor that inhibits cytotoxic T lymphocyte function and suppresses FasL expression and TNF-α secretion, has emerged as the most promising systemic therapy [4,17,18]. Multiple retrospective studies have demonstrated favorable outcomes with cyclosporine at doses of 2.5–5 mg/kg/day for 7–10 days, with standardized mortality ratios (SMR) consistently around 0.42 compared to SCORTEN-predicted mortality, indicating that observed mortality was less than half of expected mortality [3]. In contrast, IVIg monotherapy has not demonstrated a comparable mortality benefit [3,19].

This study presents two cases from opposite ends of the SJS/TEN spectrum, with contrasting severity and management. We aim to describe their clinical course, management, and outcomes, and to highlight the importance of early recognition, prompt withdrawal of the causative agent, transfer to a specialized unit, and multidisciplinary supportive care.

## 2. Materials and Methods

The study patients were admitted to the Burn Unit of the Sf Spiridon County Emergency Hospital in Iasi. Both patients included as study participants provided written informed consent for treatment and data use. The study received approval from the institutional Ethics Committee (No. 23/05.03.2026), in accordance with the principles of the Declaration of Helsinki.

### 2.1. Case Report 1

A 47-year-old male, non-smoker, non-alcohol consumer, working in construction in Ireland, with systemic lupus erythematosus (SLE) and lupus nephritis diagnosed in 2024, presented with severe mucocutaneous involvement. In early December 2025, his SLE treatment in Ireland was initiated with hydroxychloroquine (Plaquenil) 200 mg twice daily, mycophenolate mofetil 500 mg twice daily, and prednisone 4 mg daily. Approximately one week after treatment initiation, the patient developed prodromal symptoms including fever and malaise, followed by cutaneous eruptions on the trunk that appeared shortly before his departure. On December 12, he began travelling by car from Ireland to Romania despite these lesions, which worsened during the journey, with progressive pruritus followed by burning sensations affecting the soles, palms, and subsequently the face (Figure 1).

These symptoms progressed during the journey (Figure 2).

The exacerbation of symptoms prompted presentation to a local hospital, from where he was transferred through several healthcare facilities, ultimately arriving at the Burn Unit on the 18th day of the disease’s progression.

#### 2.1.1. Clinical Examination

Clinical examination on admission revealed severe mucocutaneous involvement, with detached or detachable epidermis (Nikolsky-positive) affecting approximately 35% of the body surface area (BSA), consistent with toxic epidermal necrolysis (>30% detachment). Additional erythematous and edematous changes, including facial edema, were present but were not included in the detached/detachable BSA estimate. The patient was febrile (38.3 °C). At the cephalic extremity, there was intense global facial edema with extensive hemorrhagic crusting and areas of epidermal detachment involving approximately 7% BSA, particularly perioral and perinasal, and labial micro-hemorrhagic foci. Bilateral conjunctival erythema was prominent.

The buccal mucosa appeared erythematous with bleeding tendency and superficial erosions on the soft palate. The cervical region showed epidermolysis and desquamation, moderately exudative with poorly demarcated margins.

The thoracic examination revealed multiple zones of epidermal necrosis with imprecise delimitation, tending to coalesce into plaques, occupying almost the entire anterior thorax (7% BSA) and posterior trunk (13% BSA). The shoulders and arms bilaterally showed 4% BSA involvement. Palmar and plantar surfaces (2% each) demonstrated bullae containing serous fluid, predominantly in pressure-bearing areas. These central necrotic areas, notable for absence of pain sensation, were bordered by hyperemic zones that were intensely painful spontaneously and on mobilization, with significant exudation (Figure 3).

#### 2.1.2. Laboratory Findings

Laboratory findings revealed bicytopenia with severe leukopenia (nadir 1.77 × 10^9^/L at initial presentation) and normochromic anemia (Hb 12.7 g/dL), and evidence of renal involvement (creatinine 1.31 mg/dL, urea 100 mg/dL) (Table 1). Blood cultures identified Pseudomonas aeruginosa, establishing the diagnosis of sepsis with cutaneous origin. Additionally, creatine kinase elevation indicated rhabdomyolysis, and hypoproteinemia with hypoalbuminemia was documented. Chest radiography showed no pulmonary involvement. Skin biopsy performed on the 19th day of the disease’s progression revealed partial superficial epidermal necrosis with intra- and sub-corneal bulla formation, compatible with toxic epidermal necrolysis.

Days indicate disease progression (Day 1 = first day of symptom onset, before departure from Ireland). The patient was admitted to the first hospital (County Hospital) on Day 14, transferred to our Burn Unit on Day 18, and discharged on Day 32. Skin biopsy and intravenous immunoglobulin therapy were initiated on Day 19. Total hospitalization across facilities: 19 days, of which 14 days in our Burn Unit.

Reference ranges: Hemoglobin 13–17 g/dL (male); Leukocytes 4–10 × 10^9^/L; Neutrophils 2–7 × 10^9^/L; CRP < 0.5 mg/dL; Creatinine 0.7–1.2 mg/dL; Urea 15–45 mg/dL; Glucose 70–110 mg/dL.

A skin biopsy was performed on the 2nd day after admission to the Burn Unit (the 19th day of the disease’s progression).

Treatment was established by a multidisciplinary team including dermatology, infectious diseases, plastic surgery, ENT, and ophthalmology specialists. Therapy was initiated on the 19th day of the disease’s progression with intravenous immunoglobulin (PrivigenR) at 0.4 g/kg/day for 3 consecutive days (total daily dose 36 g). Systemic corticosteroid therapy for epidermal necrolysis was initiated at our Burn Unit and comprised intravenous methylprednisolone 500 mg/day and dexamethasone; the patient had been receiving low-dose prednisone (4 mg/day) as part of his chronic lupus treatment prior to presentation. Given the positive wound cultures for Pseudomonas aeruginosa and the diagnosis of cutaneous-origin sepsis, antibiotic therapy was administered, subsequently targeted to the identified organism (imipenem and clindamycin), in line with the infectious diseases consultation. Supportive care consisted of daily therapeutic baths, topical application of Vaseline and mometasone cream, nasal mucosa care with vitamin A and Vaseline cream for 7 days, and oral mucosa treatment with borax glycerin and anesthesine (per ENT recommendations). Ophthalmological consultation diagnosed autoimmune conjunctivitis, for which NetildexR and SystaneR eye drops and Vita-POS ointment were prescribed for 7 days. Additional supportive measures included thromboprophylaxis (enoxaparin 0.4 mL), analgesia (metamizole, paracetamol, nefopam), adequate hydration, enteral nutritional supplements (NutrisonR), and intravenous vitamins.

Clinical course was favorable, with progressive improvement of cutaneous lesions, resolution of fever, and gradual decrease in pain (Figure 4 and Figure 5). At the time of IVIg initiation, epidermal detachment was still progressing; stabilization of skin involvement was observed over the following days. Given the concurrent introduction of systemic corticosteroids and comprehensive supportive care, this temporal association cannot be interpreted as a specific treatment effect.

Laboratory parameters showed normalization of leukocyte count (7.52 × 10^9^/L), improvement of renal function (creatinine 0.6 mg/dL), and reduction in inflammatory markers (CRP 3.22 mg/dL). The patient was discharged after 14 days in the Burn Unit (19 days of total hospitalization across facilities) and 32 days of total disease evolution, with complete healing and no sequelae apart from post-epithelialization scarring.

Histopathological examination was performed on three cutaneous fragments. The fragment from intact perilesional skin showed atrophic epidermis. The fragment from the blister area demonstrated detachment of the stratum corneum with a cleft occupied by compact and lamellar parakeratotic keratin lamellae, fibrin masses, and zones of recent hemorrhage with isolated inflammatory elements; the epidermis was focally atrophic with discrete spongiosis, and the subjacent dermis showed reduced perivascular inflammatory infiltrate. The fragment from the area of cutaneous lysis revealed unevenly thickened epidermis with elongated epidermal ridges, numerous apoptotic keratinocytes, and extensive necrosis from the spinous layer to the stratum corneum. The thickened papillary dermis showed reactive fibrosis, congestion, and moderate perivascular inflammatory infiltrate. PAS and Giemsa stains were negative for fungal or bacterial elements. The pathologist’s note indicated zones with atrophic epidermis, without features of interface dermatitis and without thickening of the epithelial basement membrane, with minor epidermal changes in the context of SLE (Figure 6).

### 2.2. Case Report 2

A 66-year-old female with known medical history of atopic predisposition, hypothyroidism, and arterial hypertension presented to a local hospital with abdominal pain. Following imaging and biochemical investigations, acute lithiasic cholecystitis was diagnosed, and conservative management was initiated with amoxicillin–clavulanic acid (AmoxiplusR) 2 g intravenously daily by the general surgeon. The patient was discharged after 2 days with a prescription for oral amoxicillin–clavulanic acid (AugmentinR).

Three days after initiating this treatment (disease day 1), the patient developed prodromal symptoms including fever and malaise, followed by papules and pustules with heterogeneous distribution, initially affecting the oral mucosa, lower trunk, and buttocks. This prompted re-presentation to the same facility. An allergic reaction was suspected, and the penicillin-based antibiotic was discontinued and replaced with gentamicin and ceftriaxone. Abdominal ultrasound revealed vesicular plastron formation with diminished local inflammatory changes at the gallbladder, leading to the decision to postpone cholecystectomy for 6 weeks (à froid procedure).

Exacerbation of cutaneous lesions and unfavorable dynamics of biochemical parameters (rising C-reactive protein, serum creatinine, and leukocyte count) prompted transfer to a county hospital and, several hours later, to our Burns Unit.

#### 2.2.1. Clinical Examination

Clinical examination on admission to the Burn Unit revealed erythematous plaques with heterogeneous, generalized distribution across the entire body, confluent at the buttocks and lumbar region (6% BSA), with areas of epidermolysis predominantly in these zones. Additional involvement included the bilateral thighs (3% BSA), with palmar surfaces and mild oral mucosal involvement, totaling approximately 9% BSA of skin involvement, with epidermal detachment (epidermolysis) affecting a subset of this area, predominantly the buttocks and lumbar region, consistent with Stevens–Johnson syndrome (detachment < 10% BSA). The patient was febrile (39 °C), and the Nikolsky sign was positive. Perioral hemorrhagic crusts and labial hemorrhagic granulation tissue were present, intensely painful on mandibular movement. Similar areas with dermal denudation were identified focally on the scalp and as punctate lesions on the anterior thorax. Multiple bullae with serous content were observed at the periphery of epidermolysis areas. The buccal mucosa was erythematous with stigmata of recent bleeding. Generalized facial edema of mild intensity was present, with slight bilateral conjunctival erythema (Figure 7). Genital examination revealed no vulvovaginal involvement.

#### 2.2.2. Laboratory Findings

Table 2 demonstrated elevated inflammatory markers (CRP 19.87 mg/dL), leukocytosis (12.66 × 10^9^/L) with neutrophilia (11.86 × 10^9^/L), and acute kidney injury (creatinine 2.13 mg/dL, urea 87.99 mg/dL). Chest radiography showed no pulmonary involvement. Histopathological examination of the punch biopsy performed at admission (report dated 12 January 2026) revealed subepithelial vesiculobullous lesions consistent with Stevens–Johnson syndrome. Applying the ALDEN (Algorithm of Drug Causality for Epidermal Necrolysis), amoxicillin–clavulanic acid yielded a score of 3 (“possible”), supported by a compatible latency, drug intake up to symptom onset, and prompt dechallenge; the two antibiotics introduced after symptom onset were excluded as causative.

Reference ranges: Hemoglobin 12–16 g/dL (female); Leukocytes 4–10 × 10^9^/L; Neutrophils 2–7 × 10^9^/L; CRP < 0.5 mg/dL; Creatinine 0.7–1.2 mg/dL; Urea 15–45 mg/dL; Glucose 70–110 mg/dL. Days indicate disease progression (Day 1 = onset of cutaneous symptoms; drug exposure to amoxicillin–clavulanic acid began 3 days earlier). The “County Hospital” column corresponds to Day 4 (prior to transfer); Burn Unit admission occurred on Day 6. Total hospitalization: 26 days.

Treatment considerations: dermatological exam recommended intravenous immunoglobulin (PrivigenR) at 1 g/kg for 3 days with slow infusion, combined with systemic corticosteroid therapy (intravenous methylprednisolone, oral prednisone) and topical treatment with VitanolR and mometasone ointment. However, given the favorable clinical and paraclinical evolution under systemic corticosteroid therapy alone, and following a cost–benefit analysis, the decision was made to withhold IVIg treatment.

Supportive care included daily therapeutic baths, topical application of vaseline and mometasone cream, thromboprophylaxis (enoxaparin 0.4 mL), analgesia (metamizole, paracetamol, tramadol), anxiolytic therapy, adequate hydration, enteral nutritional supplements (FresubinR), oral rinse solutions, ophthalmic drops, antacids, antiemetics, and intravenous vitamins.

The clinical course was favorable with progressive improvement under corticosteroid therapy (Figure 8 and Figure 9).

Over the course of hospitalization, laboratory parameters improved, with reduction in inflammatory markers (CRP decreased to 7.19 mg/dL), normalization of renal function (creatinine 0.90 mg/dL), and stabilization of the leukocyte count. 

The patient was discharged after 26 days in the Burn Unit, with complete healing and no sequelae apart from post-epithelialization changes (Figure 10).

Histopathological examination showed epidermis with focal tegumentary ulceration associated with underlying young granulation tissue. In the adjacent epidermis, an incipient subepidermal bulla was identified with fibrinoid necrotic areas and apoptotic keratinocytes. At the dermal level, extravasated erythrocytes and moderate lymphomonocytic inflammation with rare PMNs were observed. PAS stain was negative for fungal colonies. Toluidine blue and Giemsa stains were negative. Van Gieson stain identified focal basal necrotic areas. The morphological features were consistent with a subepithelial vesiculobullous lesion, possibly Stevens–Johnson type (Figure 11).

## 3. Summary of the Two Cases

The two cases presented herein illustrate the clinical spectrum of drug-induced epidermal necrolysis and describe two different therapeutic approaches, both with favorable outcomes. Case 1 represented a severe presentation of TEN with 35% BSA involvement in a 47-year-old male with underlying systemic lupus erythematosus, complicated by sepsis and rhabdomyolysis. Case 2 represented the milder end of the spectrum, with SJS affecting 9% BSA in a 66-year-old female with acute cholecystitis as a comorbid condition.

An important diagnostic consideration in Case 1 was the differentiation between drug-induced TEN and TEN-like lupus erythematosus (TEN-like LE). TEN-like LE is a rare manifestation (prevalence approximately 0.07% among SLE patients) characterized by insidious onset over weeks, photo-distribution, minimal genital mucosal involvement, and a prolonged clinical course [20,21]. The mean duration from initial rash to epidermal detachment in TEN-like LE is approximately 40 days (range 21–62), significantly longer than drug-induced TEN, which typically progresses within hours to days [20]. Histopathologically, TEN-like LE shows vacuolar degeneration at the dermo-epidermal junction with periadnexal lymphocytic infiltrates, whereas drug-induced TEN demonstrates full-thickness epidermal necrosis with minimal inflammatory infiltrate [20]. In our patient, the acute onset (within one week of medication initiation), extensive mucosal involvement, lack of evident photo-distribution, and favorable response to drug withdrawal support the diagnosis of drug-induced TEN rather than TEN-like LE. Formal ALDEN assessment was limited by the simultaneous initiation of three lupus medications (hydroxychloroquine, mycophenolate mofetil, and prednisone) with identical exposure timing; the highest score corresponded to hydroxychloroquine (“possible”), but no single agent could be identified as the definitive culprit, and drug causality therefore remained undetermined. It should be acknowledged that some histopathological features in this case—including focally atrophic epidermis, a relatively sparse dermal inflammatory infiltrate, and the absence of interface dermatitis—are not fully typical of florid drug-induced TEN. However, the presence of full-thickness epidermal necrosis with numerous apoptotic keratinocytes, together with the acute clinical course, extensive mucosal involvement, and the pre-existing diagnosis of SLE, is most consistent with probable drug-induced epidermal necrolysis arising in a patient with lupus, although a lupus-related TEN-like process cannot be entirely excluded.

Histopathology was therefore regarded as supportive rather than independently diagnostic, and the final diagnosis rested on clinicopathological correlation.

The causative agents differed substantially between the two cases. In Case 1, the patient had recently been initiated on triple therapy for SLE (hydroxychloroquine, mycophenolate mofetil, and prednisone), making identification of the specific culprit drug challenging. All three medications have been implicated in SJS/TEN, though hydroxychloroquine and mycophenolate mofetil carry higher reported risks [2,6]. Notably, patients with SLE are at increased risk for SJS/TEN, which may reflect both the underlying immune dysregulation and the greater number of medications prescribed to these patients [2]. In Case 2, the temporal relationship between amoxicillin–clavulanic acid initiation and symptom onset (latency period of approximately 3 days) strongly suggested this antibiotic as the causative agent, consistent with the known moderate risk associated with penicillin derivatives [6,11]. Given that aminopenicillins are also a classic trigger of acute generalized exanthematous pustulosis (AGEP), and that the initial eruption included pustular elements, AGEP was considered in the differential diagnosis. However, it was excluded on histopathological grounds: the biopsy demonstrated a subepidermal bulla with apoptotic keratinocytes and fibrinoid necrosis, rather than the subcorneal or intraepidermal spongiform pustules with neutrophilic infiltrate that characterize AGEP [3]. Regardless of the specific culprit, identification and lifelong avoidance of the offending drug and structurally related agents are essential to prevent recurrence, which may be more severe than the initial reaction [22]. More broadly, severe cutaneous adverse reactions encompass a spectrum that also includes AGEP and drug reaction with eosinophilia and systemic symptoms (DRESS), which must be distinguished from SJS/TEN on clinicopathological grounds [23].

Laboratory findings also differed markedly between the two patients. Case 1 presented with severe leukopenia (1.77 × 10^9^/L), likely related to both the underlying SLE and immunosuppressive therapy, which may have influenced both the severity of the cutaneous reaction and the inflammatory response.

In contrast, Case 2 presented with leukocytosis (12.66 × 10^9^/L) and significantly elevated CRP (19.87 mg/dL), reflecting both the acute inflammatory response to SJS and the concurrent cholecystitis. Both patients developed acute kidney injury, which normalized during hospitalization (Table 3 and Table 4). Both patients also exhibited ocular involvement, consistent with the frequent ocular mucosal disease in SJS/TEN. Notably, the atopic predisposition present in Case 2 has been associated with more severe acute ocular involvement in this setting [24].

These two patients differed substantially in age, sex, underlying disease, comorbidities, culprit drugs, and disease severity, and therefore do not constitute comparable treatment groups. Notably, the decision to withhold IVIg in Case 2 was itself driven by the milder disease course and favorable early response to corticosteroids; consequently, any difference in outcome between the two cases reflects confounding by indication and baseline differences rather than a treatment effect. The two cases are therefore presented descriptively, without any comparative or causal inference regarding the relative efficacy of the therapeutic strategies employed.

Dates are approximate for arrest of progression and re-epithelialization (~), estimated from clinical records. Length of stay is influenced by non-dermatologic factors (in Case 2, concurrent acute cholecystitis with planned interval cholecystectomy; in Case 1, sepsis and rhabdomyolysis) and does not represent a measure of cutaneous healing.

SLE—Systemic Lupus Erythematosus; TEN—Toxic Epidermal Necrolysis; SJS—Stevens–Johnson Syndrome; IVIg—Intravenous Immunoglobulin; HTN—Hypertension; BSA—Body Surface Area.

## 4. Discussion

### 4.1. Clinical Course and Outcomes of the Two Cases

The two patients illustrate opposite ends of the SJS/TEN spectrum. Case 1 presented with extensive TEN complicated by SLE and sepsis and was managed with corticosteroids and IVIg; Case 2 presented with limited SJS attributed to amoxicillin–clavulanic acid and was managed with corticosteroids alone. Both were managed according to the same core principles—prompt withdrawal of the culprit drug, transfer to a burn unit, and multidisciplinary supportive care—and both achieved complete re-epithelialization. Because the two patients differed substantially in every relevant baseline variable, their clinical courses cannot be compared, and no inference about the relative efficacy of the two regimens can be drawn.

Any differences observed between the two cases cannot be attributed to a specific treatment. Multiple factors distinguish them, including the younger age of Case 1 (47 vs. 66 years), the different underlying pathophysiology, and the concurrent management of comorbidities.

### 4.2. Role of IVIg

The use of intravenous immunoglobulin in SJS/TEN remains one of the most debated topics in dermatologic therapeutics. The theoretical rationale for IVIg is based on the demonstration that antibodies in pooled human IVIg can block the Fas (CD95) receptor on keratinocytes, thereby interrupting Fas-FasL-mediated keratinocyte apoptosis in vitro [6,11].

Initial uncontrolled reports and small case series were promising, but larger controlled studies and meta-analyses have not confirmed a consistent survival benefit for IVIg [3,19].

However, larger controlled studies have yielded conflicting results. The EuroSCAR study, the largest retrospective analysis in this field involving 281 patients from France and Germany, found that IVIg did not improve mortality compared with supportive care alone [6]. The authors concluded that “IVIg is not the best treatment of SJS/TEN and cannot generally be recommended” [6].

Recent meta-analyses have suggested that high-dose IVIg (≥2 g/kg total dose) may have beneficial effects in decreasing mortality [4]. This dose-dependent effect remains controversial, and the optimal dosing regimen has not been established. In our Case 1, we administered IVIg at 0.4 g/kg/day for 3 days (total dose 1.2 g/kg), which is lower than the high-dose protocols used in some studies reporting favorable outcomes.

Notably, a recent systematic review and meta-analysis of 43 studies with 58 treatment comparisons found no favorable effect of IVIg monotherapy compared to supportive care (mortality RR 1.25, 95% CI 0.93–1.69) [19]. However, the combination of corticosteroids plus IVIg demonstrated statistically significant benefit over IVIg alone for mortality and over corticosteroids alone for time to complete re-epithelialization [19]. Furthermore, head-to-head comparison of cyclosporine versus IVIg showed a statistically significant difference favoring cyclosporine (RR 0.18, 95% CI 0.05–0.58) [19]. These findings derive from aggregate data and cannot be extrapolated to individual patients; a report of two non-comparable cases cannot resolve the ongoing controversy regarding the role of IVIg in SJS/TEN.

It is important to note that an effective treatment should demonstrate benefit in severely affected patients with high mortality risk, rather than showing low mortality only in low-risk patients such as young patients with limited skin detachment [6]. The favorable outcome in our Case 1, despite severe disease and multiple comorbidities, is notable but cannot establish causation.

### 4.3. Corticosteroid Controversy

The role of systemic corticosteroids in SJS/TEN management has evolved considerably over time. As outlined above, older data raised concerns that corticosteroid treatment might increase the risk of infection and mortality [4,16]; however, this observation predates modern supportive care and was not confirmed in the larger EuroSCAR analysis, in which neither corticosteroids nor IVIg significantly affected mortality compared with supportive care alone [6].

However, more recent evidence has challenged this view. European multicenter retrospective studies and meta-analyses have suggested beneficial effects, particularly when high-dose corticosteroids are administered early in the disease course [4]. The Japanese guidelines for SJS/TEN, published in 2016, recommend early initiation of systemic corticosteroid therapy as first-line treatment, with IVIg or plasmapheresis added if clinical symptoms are severe or refractory [4].

The timing of corticosteroid administration appears crucial. The efficacy of systemic therapy may depend on the disease phase: in the acute phase, immunosuppressive treatments are considered suitable since strong inflammation occurs; however, at the peak period during which wide skin detachment develops, strong immunosuppressive treatment may impair re-epithelialization and increase infection risk [4].

In our cases, both patients received systemic corticosteroids. Case 1 received methylprednisolone and dexamethasone in combination with IVIg, while Case 2 was managed with corticosteroids alone (methylprednisolone and prednisone). Both achieved complete healing without significant infectious complications despite Case 1 having documented sepsis at presentation. This may reflect the importance of concurrent targeted antibiotic therapy and meticulous supportive care.

### 4.4. Confounding Factors

Several factors limit any interpretation of the two cases as a comparison.

Age difference: Case 1 was 47 years old while Case 2 was 66 years old. Age is a well-established prognostic factor in SJS/TEN, included in the SCORTEN severity score, with patients ≥40 years having worse prognosis [6].

Disease severity: The significant difference in BSA involvement (35% vs. 9%) represents fundamentally different disease severities. TEN requires more intensive wound care and carries higher risks of complications than SJS.

Underlying conditions: Case 1 had SLE, which may have altered the immune response and affected disease course. The concurrent immunosuppressive therapy for SLE may have had unpredictable effects on the cutaneous reaction. Case 2 had acute cholecystitis requiring ongoing management, which may have prolonged hospitalization independently of the SJS.

Concurrent infections: Case 1 had documented Pseudomonas aeruginosa sepsis requiring targeted antibiotic therapy, while Case 2 had no documented systemic infection. The presence of sepsis typically worsens prognosis in SJS/TEN.

Treatment differences: Beyond IVIg, other treatment differences may have influenced outcomes, including specific corticosteroid regimens, timing of drug withdrawal, and supportive care measures.

### 4.5. Limitations

This study has several important limitations inherent to case reports and small case series:

First, with only two patients, no statistical comparisons can be made, and the findings cannot be generalized. Any apparent difference in outcome between the two cases may be coincidental and cannot be attributed to a specific treatment.

Second, the retrospective nature of our analysis introduces potential biases in data collection and interpretation.

Third, the lack of randomization means that treatment selection was based on clinical judgment, introducing selection bias. Case 1 received IVIg partly due to disease severity, while Case 2 did not receive IVIg due to a favorable response to corticosteroids alone, making direct comparison problematic.

Fourth, the causative drug in Case 1 remains undetermined among three simultaneously initiated medications, limiting conclusions about drug-specific reactions.

Fifth, long-term follow-up data are not available. While both patients achieved complete epithelialization at discharge, late sequelae—particularly ocular complications, which may develop or progress months to years after the acute episode—could not be assessed, and long-term ophthalmologic follow-up is recommended [6,25]. Sixth, complete data on systemic corticosteroid dosing schedules and tapering regimens could not be fully retrieved retrospectively, which limits detailed characterization of the treatment protocols.

SCORTEN scores at Burn Unit admission were 3 for Case 1 (predicted mortality 35.3%) and 2 for Case 2 (predicted mortality 12.1%) (Table 4). Both patients survived. As SCORTEN is a validated tool for mortality prediction rather than for assessing treatment response, survival better than predicted should not be interpreted as evidence of treatment efficacy, particularly given the small number of cases; SCORTEN is reported here solely as a baseline measure of disease severity.

Despite these limitations, detailed case reports remain valuable in rare diseases like SJS/TEN, where randomized controlled trials are difficult to conduct. As noted in a recent Cochrane review, only nine studies with 308 total participants met criteria for systematic analysis, highlighting the paucity of high-quality evidence in this field [26].

## 5. Conclusions

Stevens–Johnson syndrome and toxic epidermal necrolysis represent severe, potentially life-threatening drug-induced mucocutaneous reactions that require prompt recognition, immediate withdrawal of the causative agent, and multidisciplinary supportive care.

Our study demonstrates that favorable outcomes can be achieved in both severe TEN and milder SJS with appropriate management, even in the presence of significant comorbidities and complications such as sepsis. Complete re-epithelialization was achieved in both patients without major long-term sequelae within a hospitalization period of two to four weeks.

Because the two patients differed substantially in age, disease severity, underlying conditions, culprit drugs, and concurrent complications, no comparison of their clinical courses can support inferences about the relative efficacy of the two treatment strategies; accordingly, no valid comparison of outcomes between the two cases can be made.

Treatment decisions must be individualized based on disease severity, patient characteristics, comorbidities, and available resources. Comprehensive supportive care—including wound management, fluid and electrolyte balance, nutritional support, pain control, thromboprophylaxis, and prevention of secondary infections—remains the cornerstone of SJS/TEN management regardless of the systemic immunomodulatory therapy employed. The optimal systemic treatment of SJS/TEN remains unresolved, and prospective controlled trials in comparable patients are needed to define the role of combined IVIg–corticosteroid therapy in epidermal necrolysis [19]. The principal educational value of this report lies in two aspects: the diagnostic challenge of distinguishing drug-induced TEN from TEN-like lupus erythematosus in a patient with newly diagnosed SLE receiving multiple concurrent medications, and the feasibility of a favorable outcome in severe TEN complicated by sepsis and rhabdomyolysis when core supportive principles are applied—early recognition, immediate withdrawal of the culprit drug, prompt transfer to a specialized unit, and multidisciplinary supportive care—rather than any specific systemic immunomodulatory strategy.

## Figures and Tables

**Figure 1:**
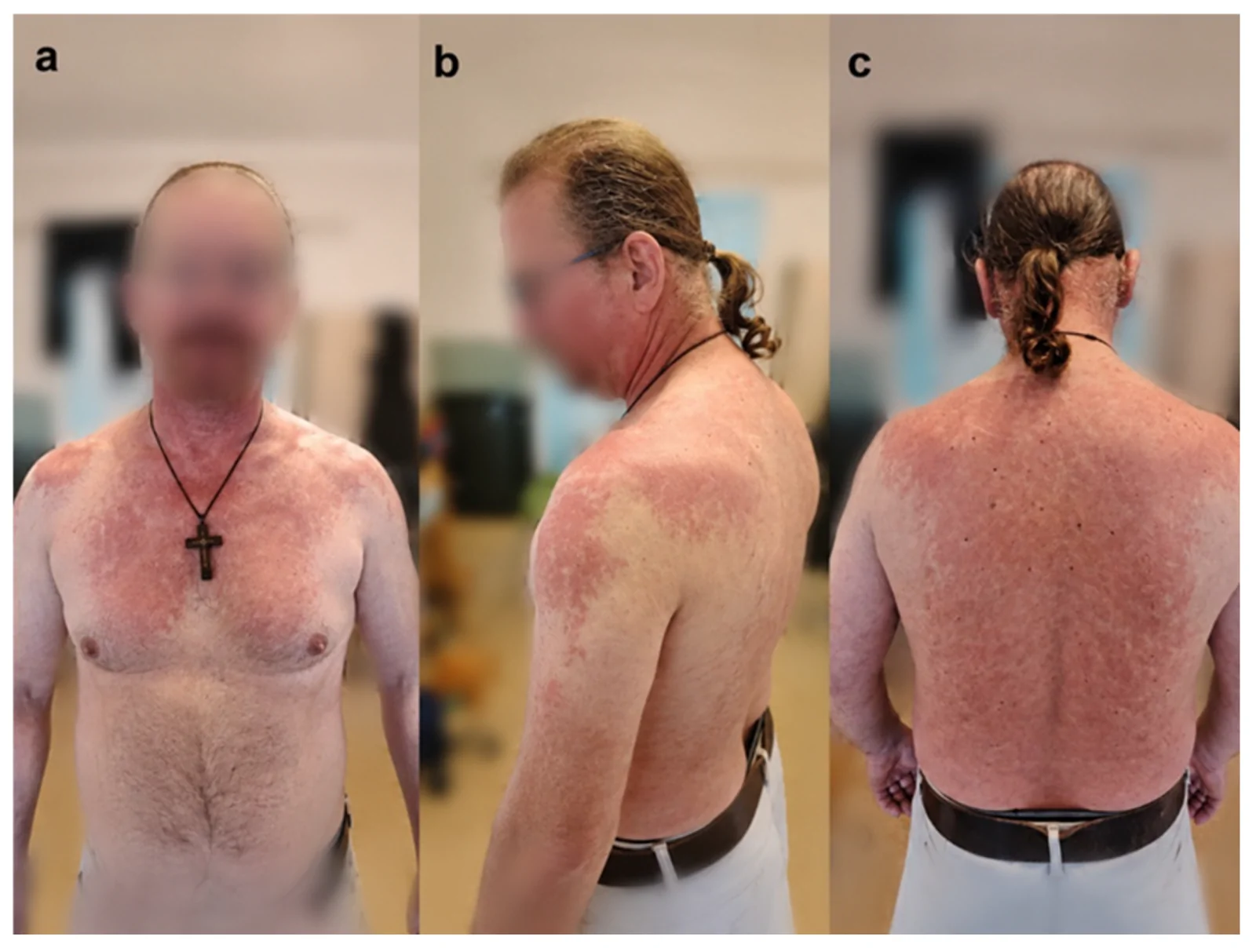
Prodromal phase (disease day 1). (**a**) Anterior view showing diffuse erythematous patches on the chest. (**b**) Lateral view with involvement of the shoulder and upper back. (**c**) Posterior view showing confluent erythema of the back.

**Figure 2:**
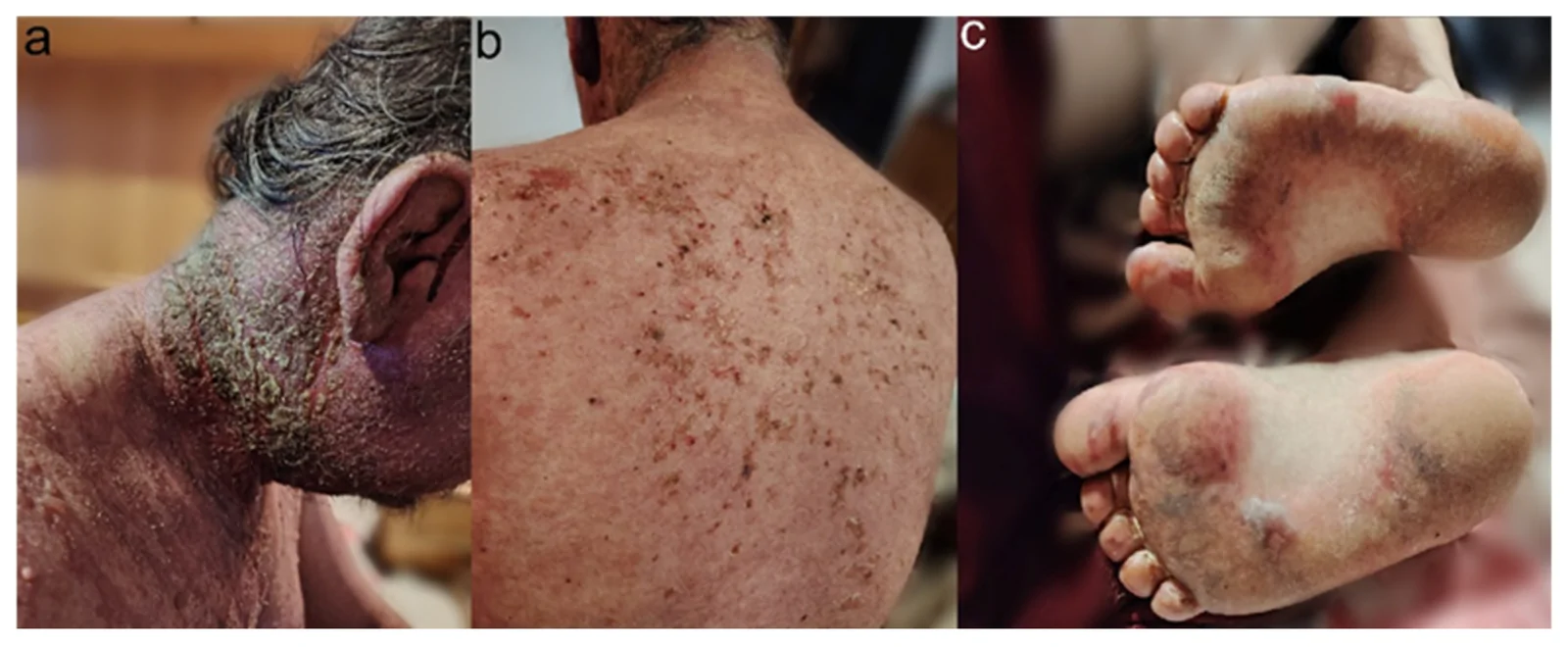
Disease progression (disease day 8). (**a**) Cervical region with early epidermolysis and crusting. (**b**) Posterior trunk with scattered erosions and crusting. (**c**) Plantar surfaces with serosanguineous bullae.

**Figure 3:**
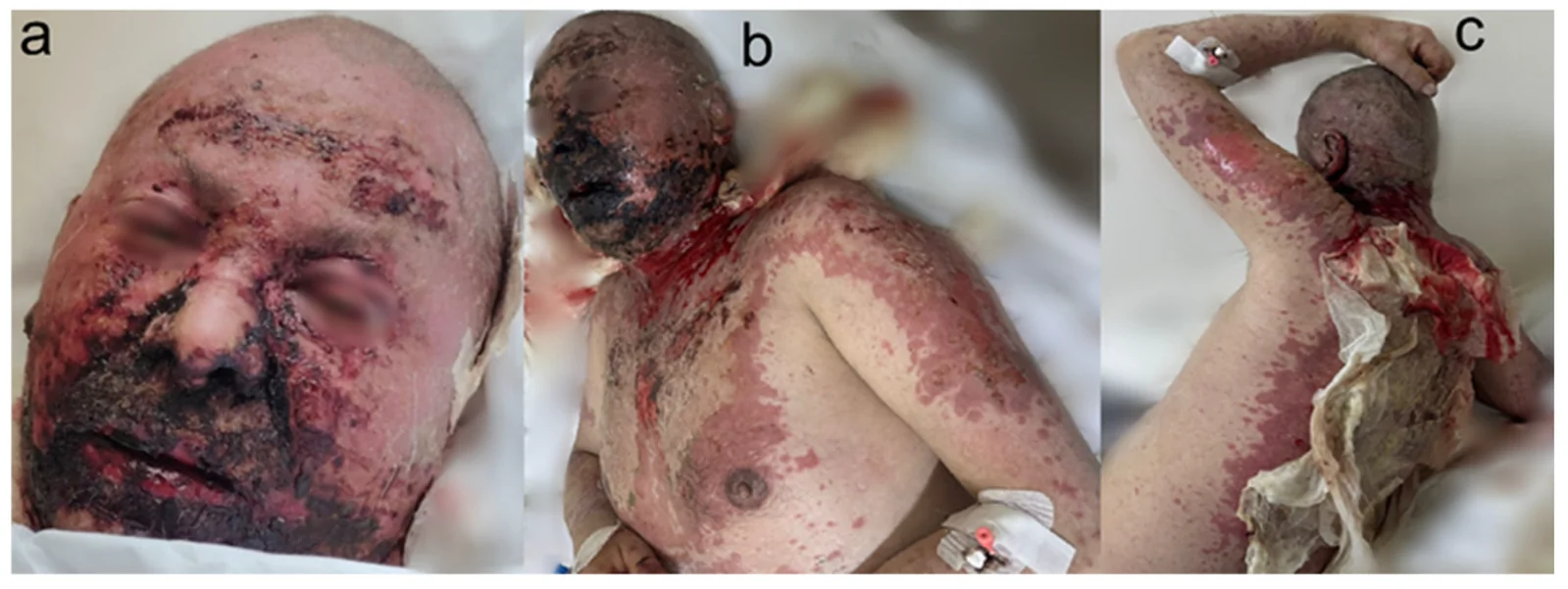
Peak severity (disease day 17, one day before Burn Unit transfer). (**a**) Frontal view of the face with severe edema and extensive hemorrhagic crusting. (**b**) Lateral view of the face, hemorrhagic latero-cervical region, and left hemithorax with confluent epidermal necrosis. (**c**) Left shoulder and posterior trunk with widespread epidermal detachment.

**Figure 4:**
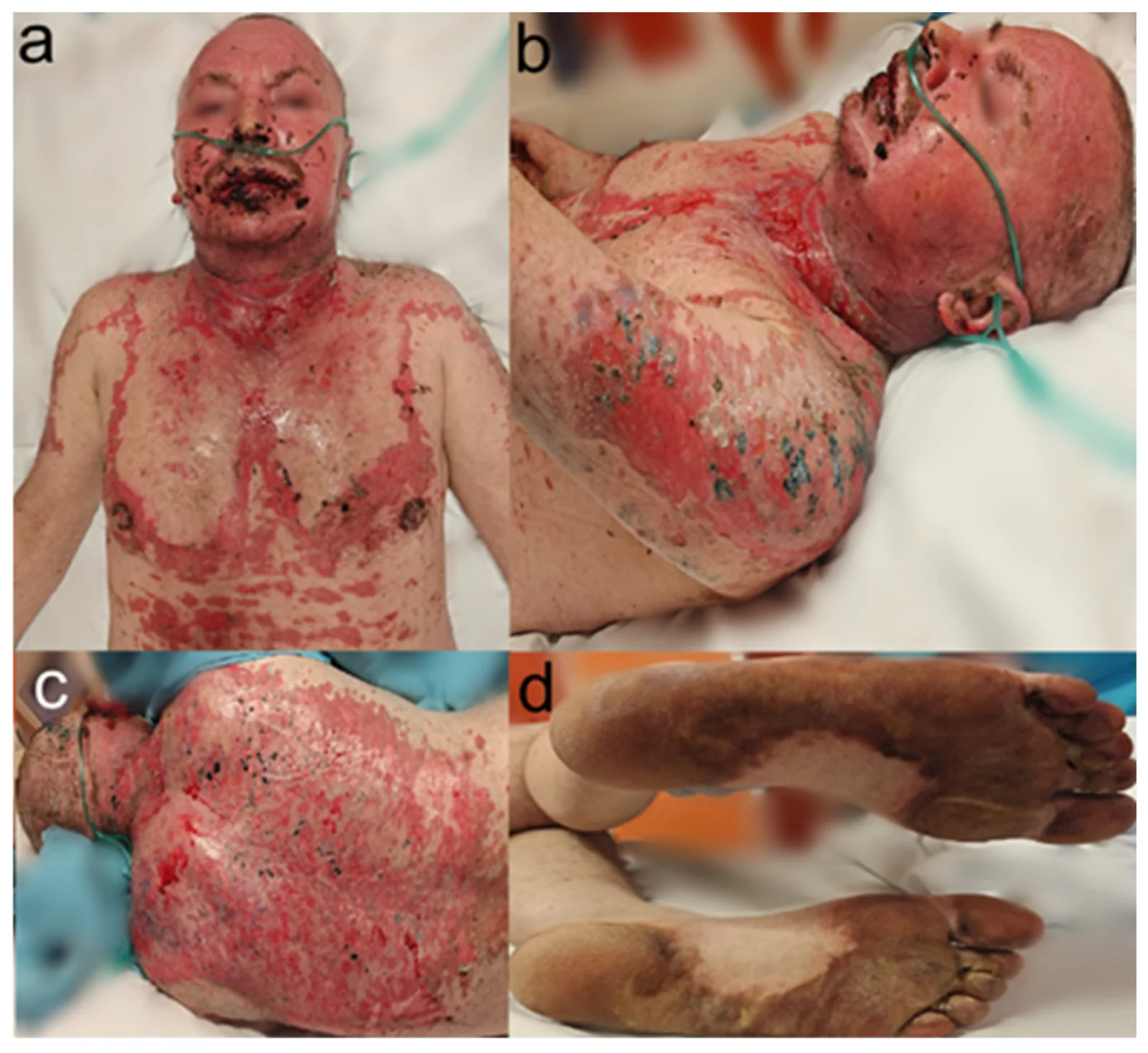
Active phase during Burn Unit treatment (disease day 24, 7th day in Burn Unit). (**a**) Face and anterior trunk with extensive epidermal necrosis and hemorrhagic crusting. (**b**) Lateral view showing involvement of the face, neck, and lateral trunk with areas of detachment. (**c**) Posterior trunk with extensive erythema and early re-epithelialization. (**d**) Plantar surfaces with persistent bullous and erosive lesions, similar to earlier stages.

**Figure 5:**
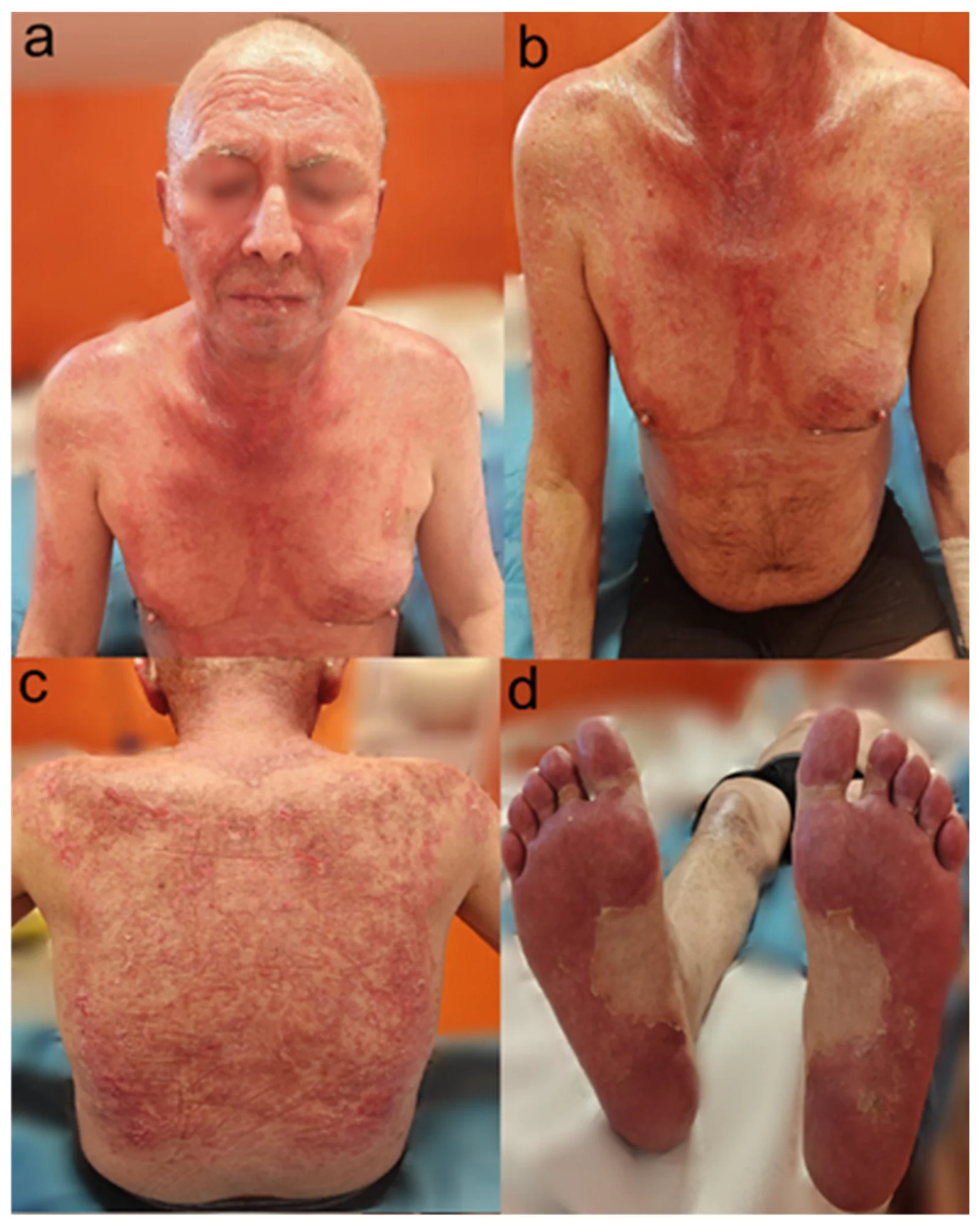
Healing phase (disease day 29, 12th day in Burn Unit). (**a**) Face with residual post-inflammatory erythema and near-complete healing. (**b**) Anterior trunk with re-epithelialization and residual erythema. (**c**) Posterior trunk with near-complete re-epithelialization. (**d**) Plantar surfaces with re-epithelializing erosions.

**Figure 6:**
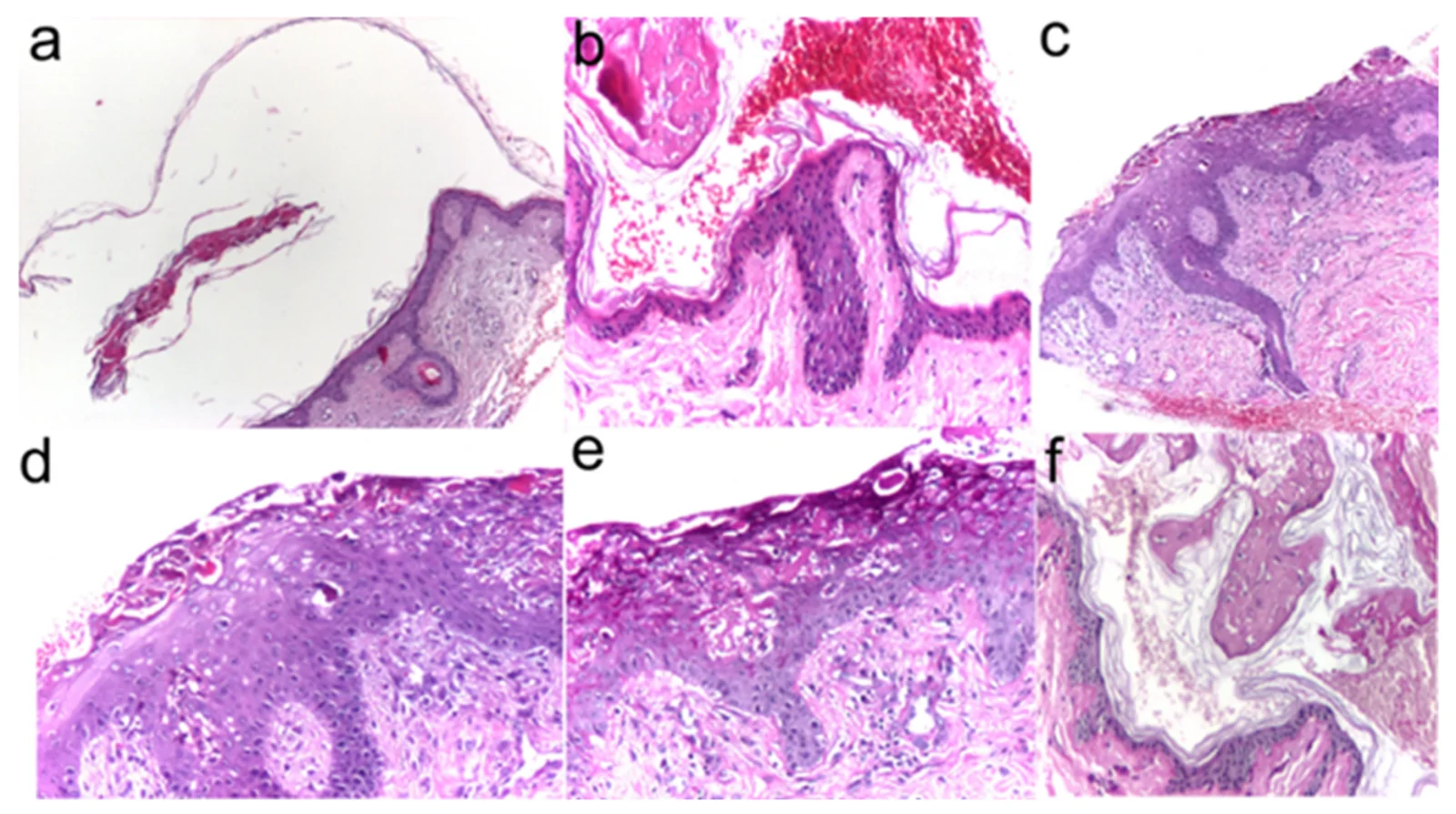
Histopathological findings (TEN). (**a**) Intracorneal bulla containing fibrin masses (HE, ×4). (**b**) Higher magnification showing fibrin, extravasated erythrocytes, and sparse inflammatory cells (HE, ×10). (**c**) Partial epidermal necrosis (HE, ×4). (**d**) Detail of epidermal necrosis with apoptotic keratinocytes (HE, ×10). (**e**) Superficial epidermal necrosis (PAS, ×10). (**f**) Absence of fungal colonies (PAS, ×10).

**Figure 7:**
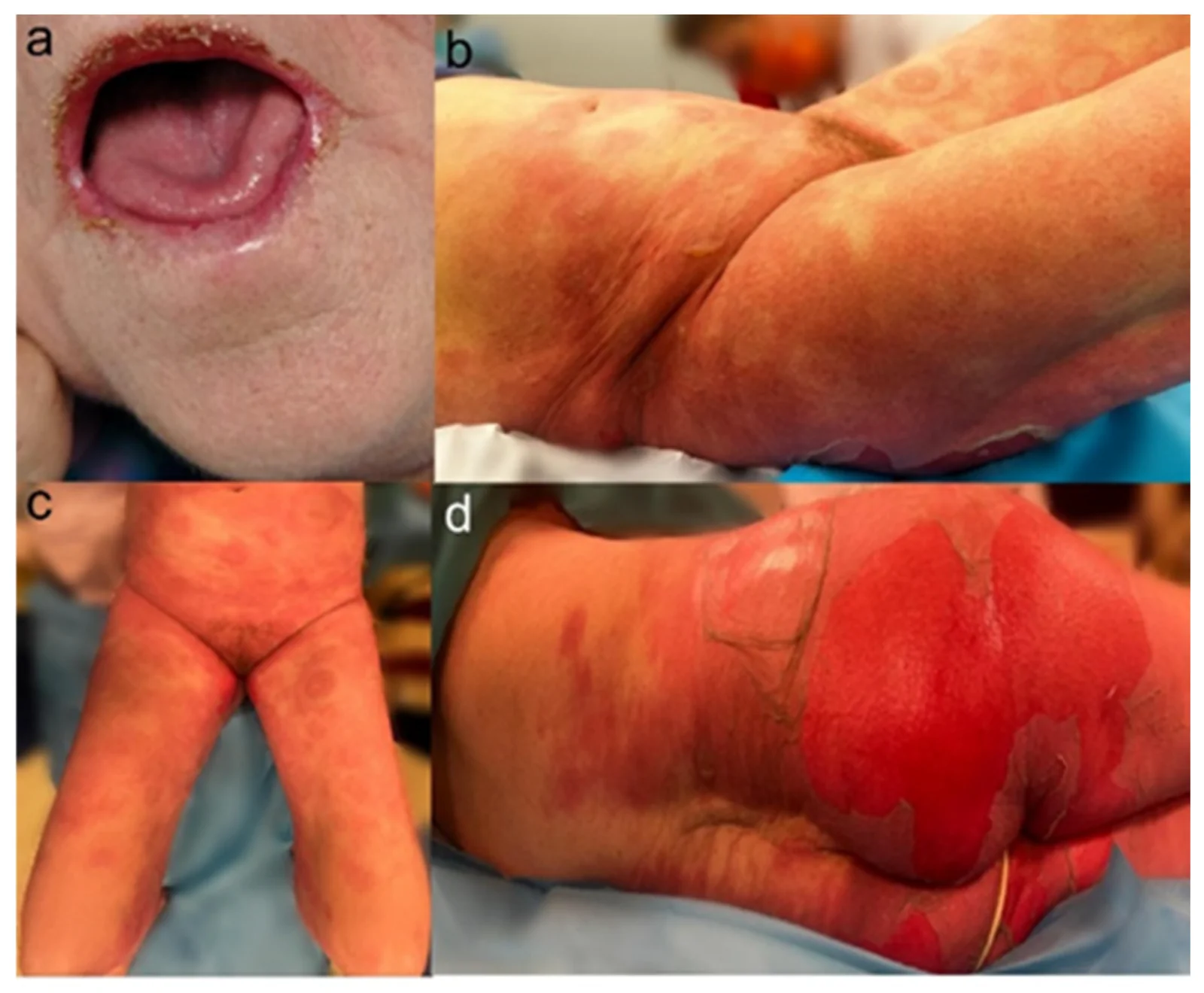
Initial presentation at Burn Unit admission (disease day 6). (**a**) Oral mucosa with erosions and hemorrhagic crusting of the lips. (**b**) Bilateral thighs and inguinal region with confluent erythematous plaques. (**c**) Anterior view of the thighs with targetoid lesions. (**d**) Buttocks and gluteal region with extensive epidermolysis.

**Figure 8:**
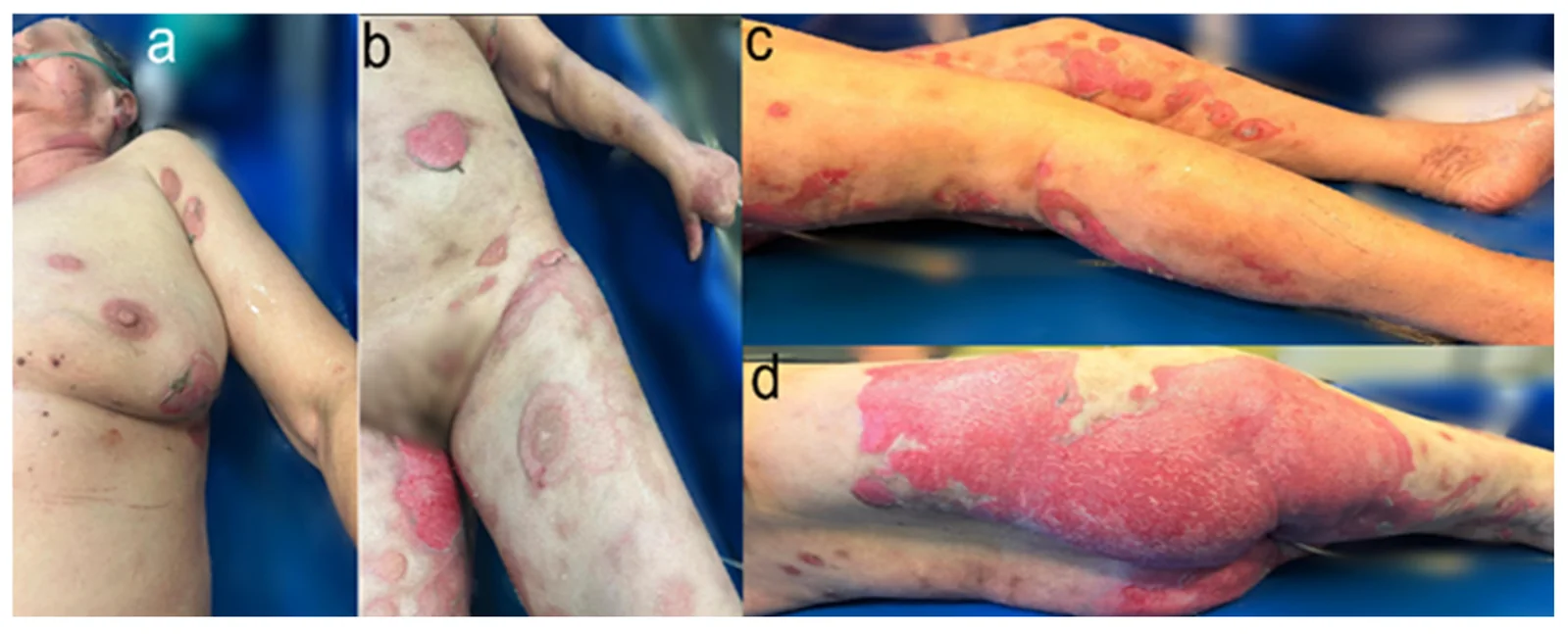
Active phase. (**a**) Anterior trunk with scattered erythematous and targetoid plaques. (**b**) Thigh with characteristic targetoid lesions. (**c**) Lower legs with erythematous plaques and erosions. (**d**) Knee and thigh with erythematous plaques and areas of epidermolysis.

**Figure 9:**
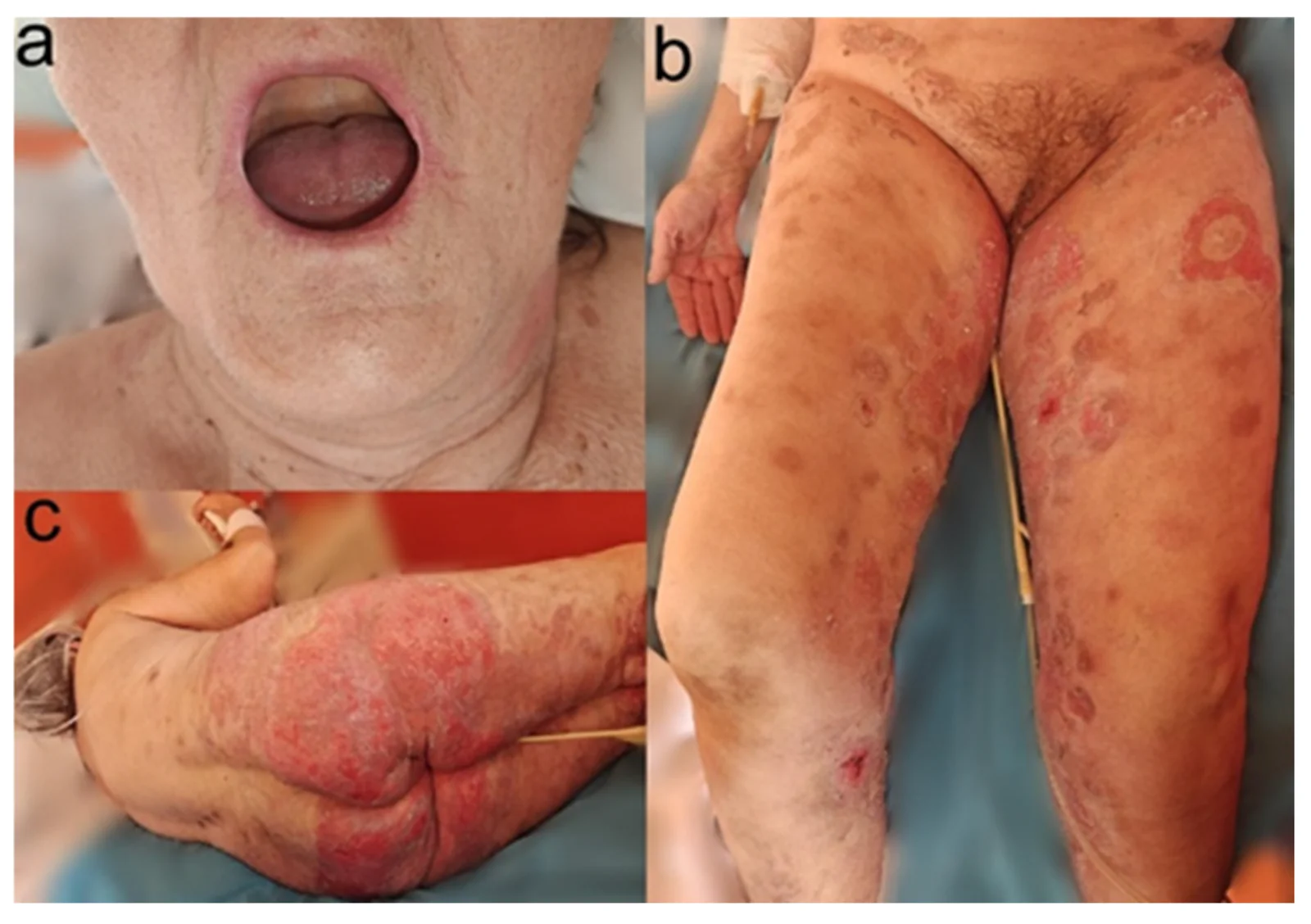
Healing phase. (**a**) Oral mucosa and perioral region with resolving erythema and crusting. (**b**) Bilateral thighs and inguinal region with post-inflammatory hyperpigmentation and residual lesions. (**c**) Hip and gluteal region with re-epithelializing erosion.

**Figure 10:**
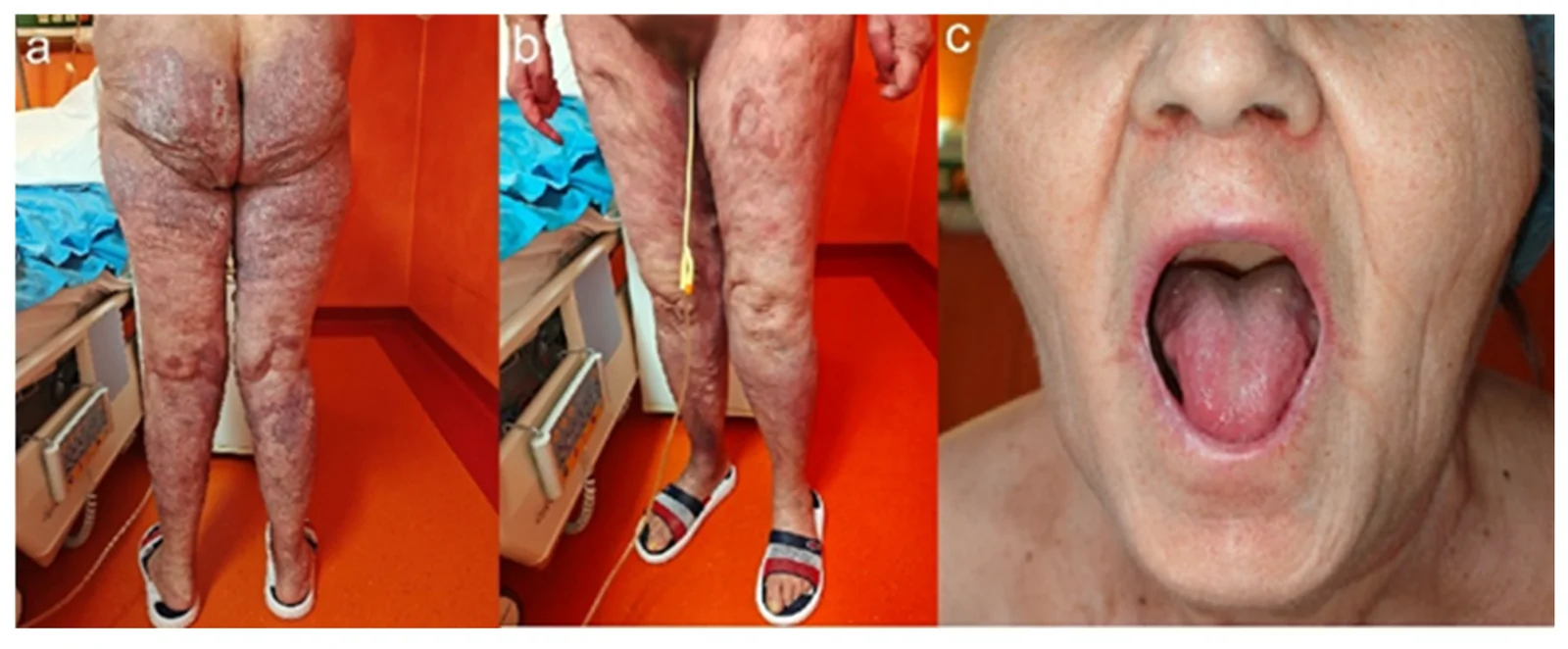
Findings prior to discharge. (**a**) Posterior view showing post-inflammatory hyperpigmentation of the buttocks and lower limbs, with complete re-epithelialization and no scarring or contractures. (**b**) Anterior view of the thighs with residual post-inflammatory changes. (**c**) Oral mucosa with residual erythema of the tongue and lips. Areas of epidermolysis at admission (9% BSA) had healed completely; residual pigmentary changes extend beyond the detached areas, corresponding to the previously erythematous regions.

**Figure 11:**
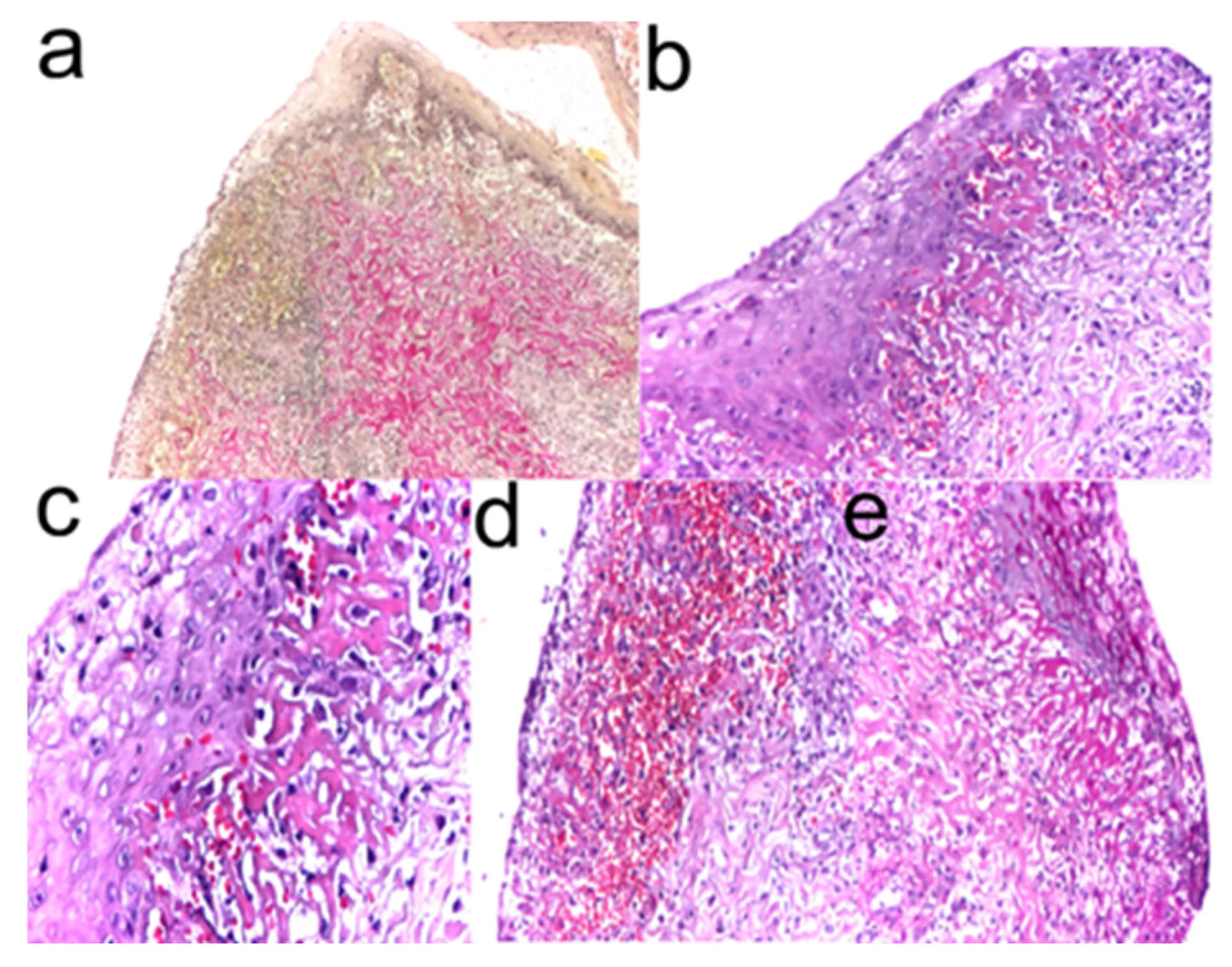
Histopathological findings (SJS). (**a**) Subepidermal bulla and tegumentary ulceration (Van Gieson, ×4). (**b**) Incipient subepidermal bulla with apoptotic keratinocytes and fibrinoid necrosis (HE, ×10). (**c**) Detail of subepidermal bulla (HE, ×20). (**d**) Ulceration with underlying young granulation tissue (HE, ×10). (**e**) Absence of fungal colonies (PAS, ×10).

**Table 1:** Laboratory Parameters.

Parameter	Day 14	Day 15	Day 17	Day 18	Day 20	Day 23	Day 28	Day 32
Hb (g/dL)	12.7	12.5	11.7	11.0	10.9	-	10.6	11.1
Leukocytes (×10^9^/L)	1.77	3.06	3.25	3.88	4.07	-	13.4	7.52
Neutrophils (×10^9^/L)	1.48	2.64	2.44	3.28	2.9	-	12.24	5.82
CRP (mg/dL)	-	7.72	4.39	-	-	1.9	3.22	-
Creatinine (mg/dL)	1.31	1.11	0.85	0.61	0.65	0.66	0.81	0.6
Urea (mg/dL)	100	118	117	63	43	31	42	64
Glucose (mg/dL)	139	144	108	160	-	90	86	83

**Table 2:** Case 2 Laboratory Parameters.

Parameter	County Hospital (Day 4)	Day 5	Day 6	Day 7	Day 8	Day 12	Day 17	Day 20
Hb (g/dL)	12.5	12.9	12.6	12.2	-	12.4	11.7	11.9
Leukocytes (×10^9^/L)	12.66	13.55	9.55	7.57	-	11.37	19.83	15.56
Neutrophils (×10^9^/L)	11.86	13.00	6.72	6.07	-	8.72	17.22	12.44
CRP (mg/dL)	19.87	16.2	-	-	7.19	-	-	-
Creatinine (mg/dL)	2.13	1.93	2.17	1.47	1.27	1.14	0.93	0.90
Urea (mg/dL)	87.99	110.78	124	104	72	84	85	88
Glucose (mg/dL)	-	-	118	104	87	93	82	68

**Table 3:** Clinical Summary of the Two Cases.

Parameter	Case 1 (TEN)	Case 2 (SJS)
Age/Sex	47 years/Male	66 years/Female
Diagnosis	TEN	SJS
BSA involvement	35%	9%
Comorbidities	SLE, lupus nephritis	Atopic predisposition, hypothyroidism, HTN, acute cholecystitis
Suspected causative drug	Hydroxychloroquine, mycophenolate mofetil, or prednisone (undetermined)	Amoxicillin–clavulanic acid
Symptom onset	13 December 2025	28 December 2025
First hospital admission	26 December 2025	29 December 2025
Culprit drug withdrawal	26 December 2025	29 December 2025
Latency (drug → onset)	~7 days	~3 days
Transfer to Burn Unit	30 December 2025	2 January 2026
Systemic treatment initiation	Corticosteroids 30 December 2025; IVIg 31 December 2025	Corticosteroids 2 January 2026 (IVIg withheld)
Arrest of detachment progression	~31 December 2025	~7 January 2026
Complete re-epithelialization	12 January 2026	~30 January 2026 (near-complete at discharge)
Complications	Sepsis (*P. aeruginosa*), rhabdomyolysis	Acute kidney injury
Treatment	IVIg (0.4 g/kg/day × 3 days) + systemic corticosteroids	Systemic corticosteroids alone (IVIg recommended but withheld)
Discharge	13 January 2026	28 January 2026
Burn Unit length of stay	14 days	26 days
Outcome	Complete healing	Complete healing

**Table 4:** SCORTEN Severity Score at Admission to the Burn Unit.

SCORTEN Parameter	Case 1 Value	Points	Case 2 Value	Points
Age > 40 years	47 years	1	66 years	1
Heart rate > 120 bpm	102 bpm	0	99 bpm	0
Malignancy	No	0	No	0
BSA > 10%	35%	1	9%	0
Serum urea > 60 mg/dL (BUN >28 mg/dL)	63 mg/dL	1	124 mg/dL	1
Serum glucose > 252 mg/dL	160 mg/dL	0	118 mg/dL	0
Serum bicarbonate < 20 mmol/L	23.3 mmol/L	0	25.7 mmol/L	0
Total SCORTEN	3		2	
Predicted mortality	35.3%		12.1%	
Actual outcome	Survived		Survived	

SCORTEN was calculated using parameters obtained within the first 24 h of admission to our Burn Unit (disease day 18 for Case 1; disease day 6 for Case 2). Serum urea was assessed against the urea-equivalent threshold of 60 mg/dL (corresponding to BUN > 28 mg/dL). Predicted mortality: 0–1 points = 3.2%; 2 points = 12.1%; 3 points = 35.3%; 4 points = 58.3%; ≥5 points = 90%.

## Data Availability

The data supporting the findings of this study are available from the corresponding author upon reasonable request. The data are not publicly available due to patient privacy restrictions.

## Abbreviations

The following abbreviations are used in this manuscript:

AGEP	Acute generalized exanthematous pustulosis
AIDS	Acquired immunodeficiency syndrome
BSA	Body surface area
BUN	Blood urea nitrogen
CI	Confidence interval
CRP	C-reactive protein
ENT	Ear, nose and throat
EuroSCAR	European Study of Severe Cutaneous Adverse Reactions
FasL	Fas ligand
Hb	Hemoglobin
HE	Hematoxylin and eosin
HIV	Human immunodeficiency virus
HTN	Hypertension
IVIg	Intravenous immunoglobulin
LE	Lupus erythematosus
NK	Natural killer
NSAIDs	Nonsteroidal anti-inflammatory drugs
OR	Odds ratio
PAS	Periodic acid–Schiff
PMNs	Polymorphonuclear neutrophils
RR	Risk ratio
SCORTEN	Severity-of-Illness Score for Toxic Epidermal Necrolysis
SJS	Stevens–Johnson syndrome
SLE	Systemic lupus erythematosus
SMR	Standardized mortality ratio
TEN	Toxic epidermal necrolysis
TNF-α	Tumor necrosis factor alpha
